# Fabricating a Raman spectrometer using an optical pickup unit and pulsed power

**DOI:** 10.1038/s41598-020-68650-7

**Published:** 2020-07-16

**Authors:** Young Chai Cho, Sung Il Ahn

**Affiliations:** Department of Chemistry Education, Graduate Department of Chemical Materials, Institute for Plastic Information and Energy Materials, Pusan National University, Busandaehakro 63-2, Busan, 46241 Republic of Korea

**Keywords:** Chemistry, Materials science

## Abstract

Although Raman spectroscopy is a major analytical tool in modern chemical experiments, commercial Raman spectrometers remain very pricey for educational and research purposes in individual university laboratories. Thus, this study focused on the structural similarity between the Raman spectrometer and an optical pickup unit (OPU), which is an inexpensive compact optical device used for a part of optical discs. The study investigated whether or not a full set of Raman spectrometer can be developed at a cost of less than 1,000 US$. The OPU-based Raman spectrometer was fabricated using 3D printer-made components, a Raman edge filter, and a laser diode with a wavelength of 520 nm as the light source. A function generator was used as a pulsed power source to analyze the characteristics of the OPU Raman spectrometer according to various frequencies and duty ratios. When using a pulsed DC power supply, the laser wavelength tended to move to a longer wavelength with increases in duty ratios. That is, the higher the frequency at the same duty ratio, the weaker the background light intensity compared with the scattered Raman signal intensity. The findings illustrate that Raman signal strength can be adjusted by adjusting the focal length of the objective lens of the OPU through an external adjustment of an additional DC power. In the Raman spectra of all solid and liquid samples used, the maximum error rate reached approximately 11 cm^−1^, whereas the maximum intensity deviation reached approximately ± 6%. The cost of the complete OPU Raman spectrometer is less than 1,100 US$ using a function generator as power source and less than 930 US$ using a DC adapter. If the optical density (OD) 6 filter can be replaced with the OD 4 filter, then the costs are expected to decrease to approximately 730 US$.

## Introduction

**Figure 1:**
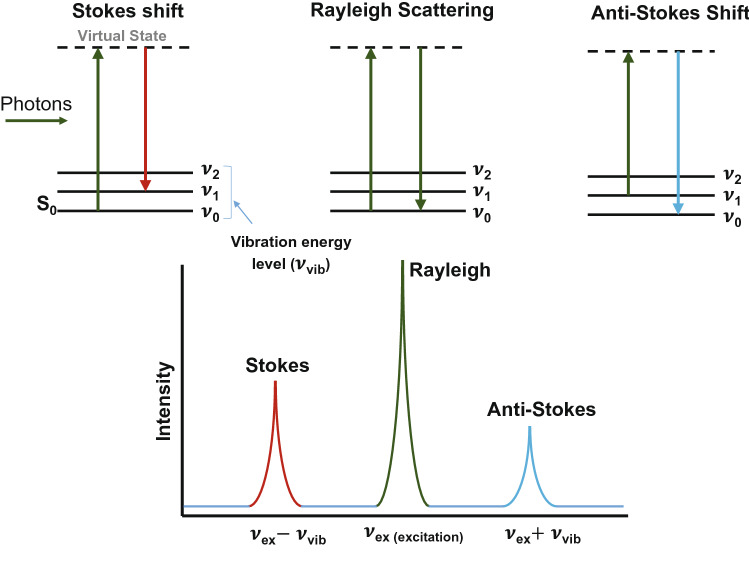
Rayleigh scattering versus Stokes Raman and anti-Stokes Raman scattering.

**Figure 2:**
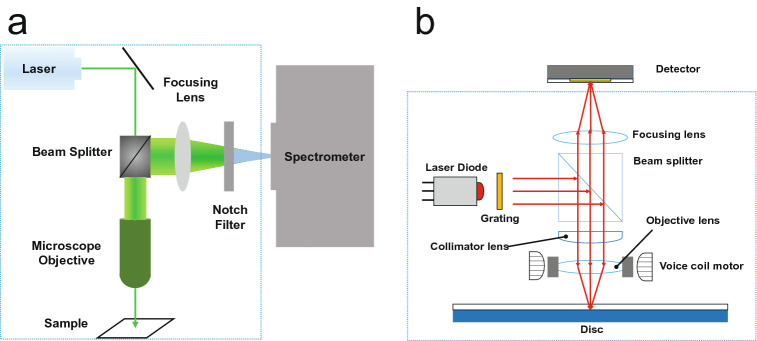
Simplified Raman spectrometer and OPU structure comparison: (**a**) Raman spectrometer. (**b**) OPU of a compact disc player.

Raman spectrometers are very powerful tools used for the simple and rapid identification of chemical species in chemical-related laboratories. In particular, it is considered one of the essential spectroscopic methods along with the research and development of low-dimensional carbon compounds, such as carbon nanotubes, graphene, and carbon dots^1–6^. Since C. V. Raman discovered Raman scattering in 1928^7^, various Raman spectrometers have been developed after extensive research and improvement. Light scattering can be classified into Mie and Rayleigh scatterings dependent on the size of scattering materials^8^. Mie scattering is a phenomenon in which the light of all colors is scattered evenly regardless of the wavelength due to particle sizes that are larger than the incident wavelengths of lights. When the size of the scattering matter is much smaller than the wavelength of light, the difference lies in the degree of scattering according to the wavelength of the incident light. The second form, namely, Rayleigh scattering, causes the sky to appear blue. Most photon incidents on a molecule undergo Rayleigh scattering at the same wavelength as the incident light. However, a few photons scatter inelastically, where the wavelength of the scattered light changes due to the interaction with the molecules. When such a Raman scattering occurs, the wavelengths of the scattered light may decrease or increase, and such a scattering is called Stokes Raman scattering or anti-Stokes Raman scattering, respectively (Fig. 1). Anti-Stokes Raman scattering consistently holds lesser intensity than Stokes Raman scattering because it is a phenomenon that occurs in molecules in the excitation state. Therefore, most Raman spectrometers are manufactured using the Stokes Raman scattering, which occurs in ground-state molecules. When Raman scattering occurs, the wavelength of the incident light shifts due to the difference in the intrinsic vibrational energy of the molecule. Each molecule has a specific vibrational energy, such that this frequency difference has its own value. Therefore, the composition or structure of a chemical can be confirmed through Raman scattered light. Figure 2a provides the structure of a simplified Raman spectrometer.

Raman spectrometers have been commercialized in various means dependent on the wavelength of laser light sources, optical paths, or slightly different arrangements in the interior space. However, commercialized Raman spectrometers are difficult to use in individual university labs or educational purposes because of their prices. To solve the cost problem, various studies have been conducted to directly manufacture a Raman spectrometer at a low price^9–12^. Mohr et al. studied a Raman spectrometer and assembled optical components and a green laser point at 532 nm applicable to undergraduate education and research purposes^9^. In addition, Rossi et al. studied a Raman spectrometer using a commercially available camera and various optical filters^12^. Although previous studies reported cheaper prices than commercially available Raman spectrometers, cost and structural problems remained due to problems in the light path control and use of expensive spectrometers.

The optical pickup unit (OPU) used in the optical disk in Fig. 2b is an extremely small and precise optical device. Many studies have applied it as an optical device, such as an optical microscope^13^, for measuring the thermal vibration of a microcantilever for atomic force microscopy^14^. The OPU is also useful for refractive index measurement at the femtoliter scale^15^ and a biological sensor at the nano- and micro-scale levels^16^ due to its various functions. It has also been applied in micropatterning devices^17^, optical fluid scanning to detect protein aggregation in microfluidics^18^, and DNA replication using magnetic nano-beads^19^. As previously described, the OPU has been applied to various optical devices and research, but it has been rarely used on a Raman spectrometer. In fact, the structure of the OPU has a large similarity with that of a Raman probe. Therefore, the present study fabricated a Raman spectrometer using the OPU to overcome the aforementioned problems, such as difficulty in controlling optical paths and cost.

## Results and discussion

**Figure 3:**
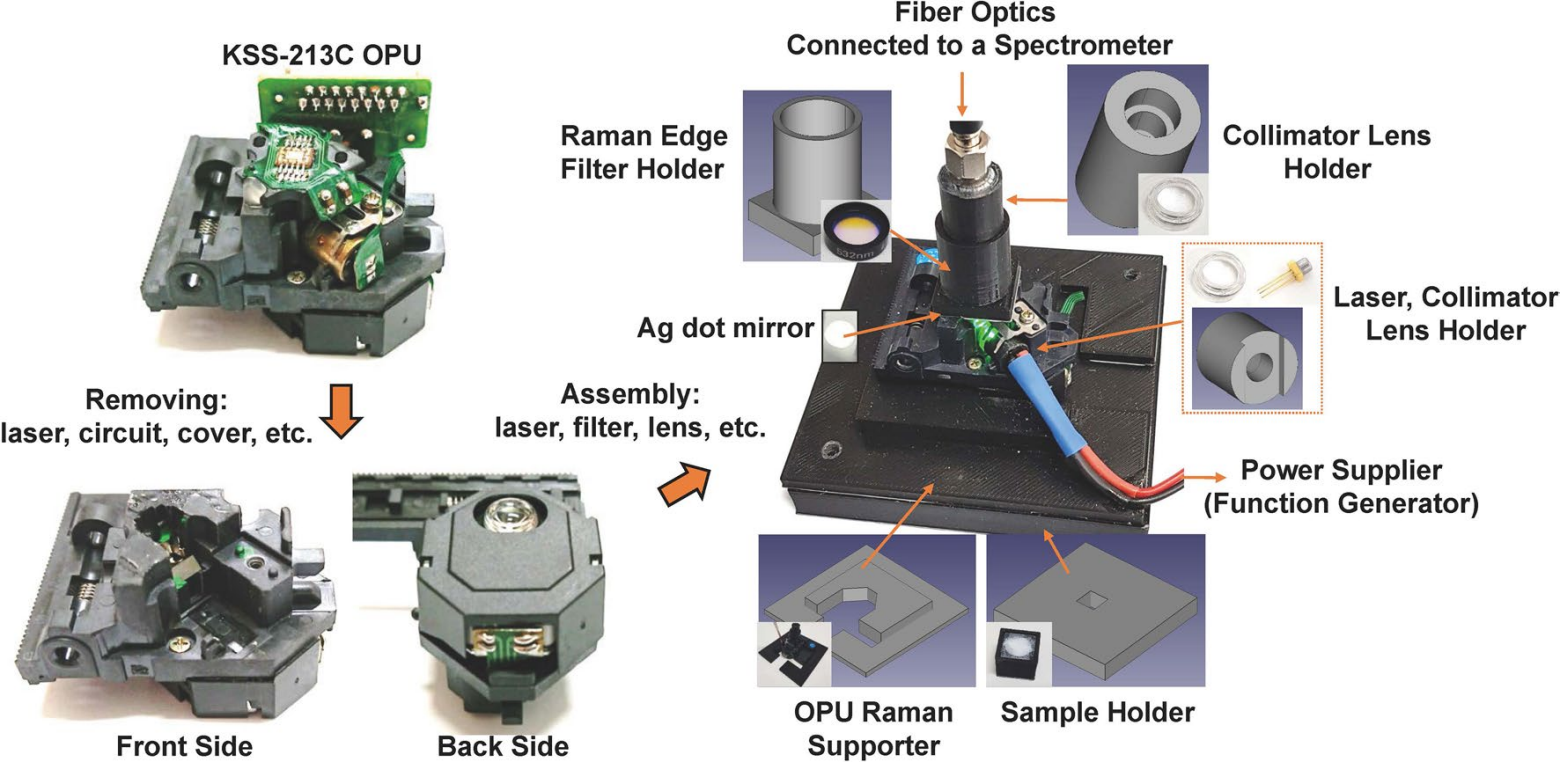
Schematics of the Raman spectrometer manufacturing process and optical images of each part.

Figure 3 shows the process of manufacturing the Raman spectrometer using the OPU (KSS-213C) for a compact disc player. Figure 3 shows that the OPU Raman was manufactured using a laser diode (LD), essential optical parts, and connecting parts made of 3D printers, while maintaining the optical path, lens, and focal length adjustment functions of the OPU. We analyzed the OPU Raman spectrometer using various electrical signals formed by the function generator as the power source (see the Experiments section and Supplementary Information for detailed descriptions of the fabrication method). The LD is frequently operated by a direct current power source. However, we controlled the electrical signal to reduce the power consumption of the LD, which leads to a reduced risk of eye damage. In addition, this feature can enhance resolution and reproducibility by reducing heat formation during the operation of LD, which causes a wavelength shift in the laser. Toward this end, the Raman spectrometer was analyzed using a square-pulsed DC made from a function generator as the power source. First, the frequency was fixed at 100 Hz to analyze the effect of the duty ratio on the OPU Raman, and the duty ratio was adjusted from 10 to 80% to analyze the shift in the center wavelength of the laser and benzene spectrum according to each duty ratio.

**Figure 4:**
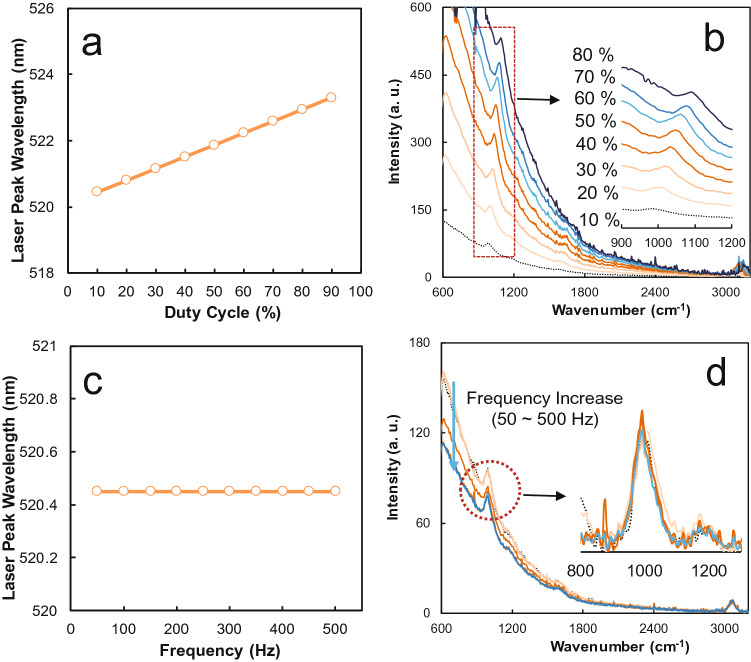
Spectral characteristics of laser and benzene according to pulsed DC signals: (**a**) Wavelength shift of LD according to duty ratio. (**b**) Raman spectra of benzene according to the duty ratio. (**c**) Wavelength shift of the LD according to the frequency at 10% duty ratio. (**d**) Raman spectra of benzene dependent on frequency at 10% duty ratio.

After the measurement, the horizontal axis indicated by the wavelength was changed in wavenumber units based on the laser wavelength. Raman spectra were acquired after baseline fitting using MagicPlot Student (ver. 2.7.2, Magicplot Systems, LLC; see Supplementary Information on how to correct the baseline). Figure 4a shows that the center wavelength of the laser increases with the increase in the duty ratio. This result is considered a phenomenon that occurs as the current amount increases, whereas the internal temperature of the LD increases with the increase in the duty ratio. Although this case differs from the LD, similar wavelength changes have been reported in light-emitting diodes in terms of the duty ratio^20^.

The graph in Fig. 4b denotes the Raman spectra of benzene obtained with the increase in the duty ratio. When comparing the position of the 992 cm^−1^ peak as the reference peak, the intensity of the peak gradually increased with the increase in the duty ratio, and the position of the peak indicated a shift similar to the wavelength shift of the laser.

To understand the characteristics of the OPU Raman according to the frequency, the duty ratio was fixed. By contrast, the frequency was varied to measure the change in the center wavelength of the LD and reference peak position of benzene. The deformation of the voltage waveform was observed at a high frequency. Thus, the frequency dependency of the OPU Raman was analyzed within a range of 500 Hz or less.

First, after setting the duty ratio to 10%, frequency was increased by 50 Hz to measure the change in the center wavelength of the laser. Figure 4c illustrates that the laser wavelength had slight changes with the increase in the frequency. In contrast to the Raman spectrum of benzene, which is dependent on the duty ratio, the main peak of benzene at 992 cm^−1^ was nearly unchanged with the increase in the frequency (Fig. 4d). However, although the position and relative intensity of the Raman peak were the same, the intensity of the Rayleigh scattering was reduced under the high-frequency conditions. These results indicate that Raman scattering intensity increases at the same laser power when measured at high frequencies at the same duty ratio.

**Figure 5:**
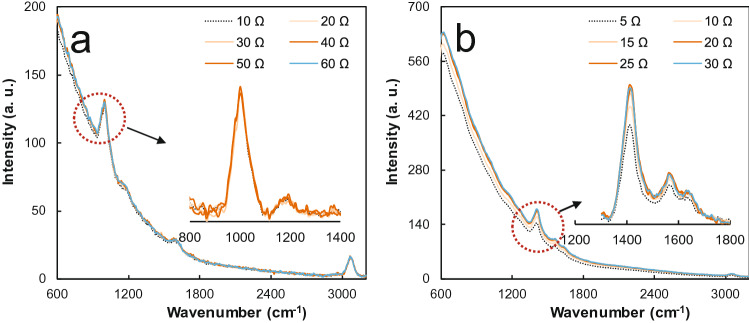
Raman spectrum obtained by adjusting focal length using a variable resistor. (**a**) Liquid benzene. (**b**) Anthracene in solid powder form.

Any device using an OPU has the advantage of focusing through vertical and horizontal distance adjustments when voltage is applied to the coil around the objective lens of the OPU. The study investigated the effect of focal length adjustment by controlling the applied voltage using a variable resistor (100 Ω) and a battery (3 V). Experiments were conducted using liquid benzene and the solid powder form of anthracene. The sample was placed at a distance similar to the known focal length of the objective lens (an aspheric lens with a focal length of 3.86 mm, working distance of 2.1 mm, and 0.45 numerical aperture), and the Raman spectrum according to resistance change was analyzed. Figure 5a shows changes in the Raman spectra of liquid benzene with the increase in resistance, where little change was observed within a certain focal length. Conversely, in the case of anthracene, a nonuniform solid sample was noted in Fig. 5b, where the intensity of the peak at 1,402 cm^−1^ varies depending on the resistance value. After passing the critical resistance (25 Ω), the figure shows that nearly the same intensity of the peak is obtained. This result shows that the focal length of the OPU Raman can be adjusted by simply adjusting the external voltage for the coil of the OPU.

**Figure 6:**
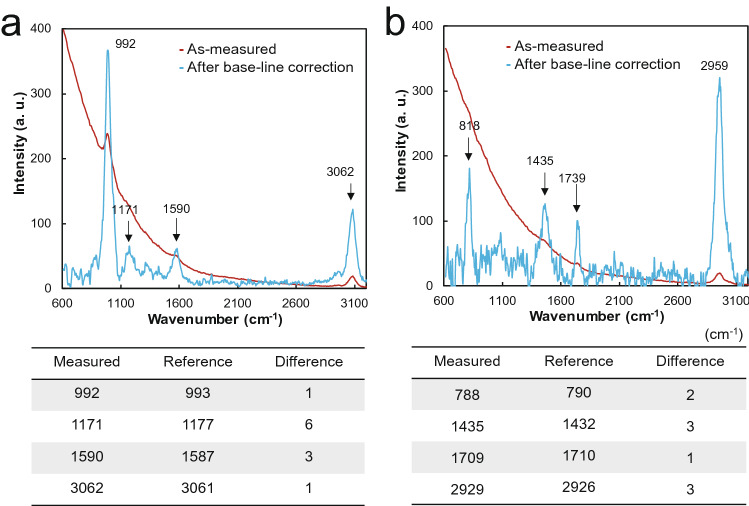
Comparison of Raman spectra and reference Raman peak of a liquid sample obtained using the OPU Raman spectrometer. (**a**) Benzene. (**b**) Acetone.

**Figure 7:**
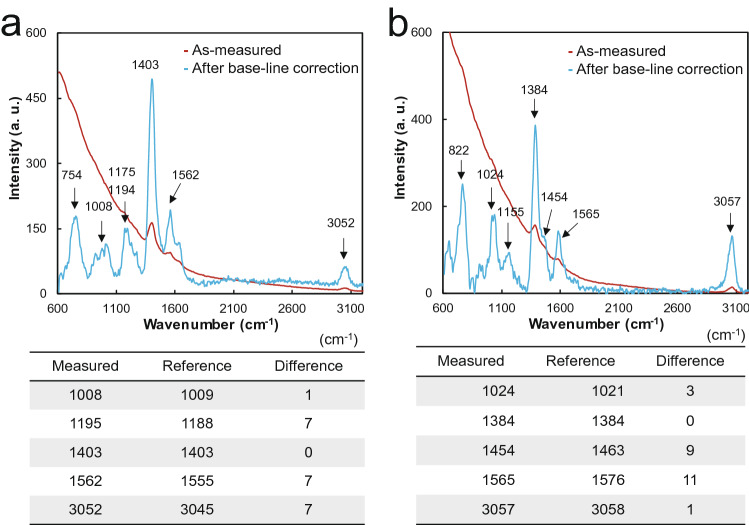
Comparison of the major peaks in the Raman spectrum and reference Raman peak of the solid samples obtained using the OPU Raman spectrometer. (**a**) Anthracene. (**b**) Naphthalene.

Considering the characteristics of the OPU Raman obtained in the basic experiment, Raman spectra were obtained for solid (anthracene and naphthalene) and liquid (benzene and acetone) samples. Afterward, Raman spectra were analyzed after a baseline correction of the spectrum for each sample. The Raman spectrum was obtained for the liquid sample under the following conditions: 400 Hz, duty ratio of 10%, and 20,000 ms integral time. Figure 6a illustrates the baseline corrected Raman spectrum of benzene, and Table 1 provides data on the major peaks of the known Raman peaks of benzene^21^. The spectrum shows a difference of up to 6 cm^−1^ compared with known Raman peaks. In the case of acetone, the Raman signal was weakly measured at a low duty ratio and under a duty cycle of 40%, 100 Hz, and 15,000 ms integral time. After measurement, a baseline correction of the Raman spectrum was carried out after the correction of the laser wavelength measured at a 40% duty ratio. A difference of up to 3 cm^−1^ was observed compared with each reference peak position in Fig. 6b^22^. For solid samples, measurements were performed under 400 Hz, duty ratio of 10%, and 6,000 ms integration time. Raman spectra were obtained and analyzed after the baseline adjustment in the same manner as the liquid samples. The spectrum for anthracene in Fig. 7a shows a difference of up to 7 cm^−1^ at each major peak compared with that of the reference^23^. In addition, naphthalene in Fig. 7b shows a positional change of up to 11 cm^−1^.

**Table 1:** Total cost of fabrication of a set of the OPU Raman spectrometer.

Parts	Supplier (or where to buy)	Price (USD)
OPU (KSS-213C)	Smartech Electronics and Machinery Manufacturing Co., LTD	4
520 nm laser (PL520)	Osram opto semiconductors	20
Raman Edge Filter (532 nm)	Edmund Optics, 12.5 mm, OD 6 or Replaceable with OD 4	445 245
Collimator lens (PMMA)	Shenzhen Tyson Tech. Co	< 1
Optical fiber (200 µm)	Thunder Optics	47
Spectrometer (R spectrometer)	Thunder Optics	350
Printed parts (PLA 500 g)	Plasil	~ 10
Function generator (AG051)	Owon or a DC adapter	199 30
Others	Variable Resistor, 1.5 V Battery, Adhesive, etc	~ 20

**Figure 8:**
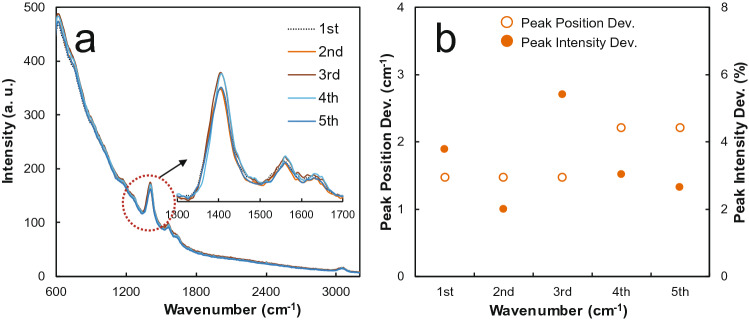
Reproduction characteristics of the OPU Raman spectrometer. (**a**) Raman spectra of anthracene according to the measurement cycle (periodic on–off of the system 10 min after each end of measurement). (**b**) Peak position and intensity deviation for each spectrum.

The reproducibility of the OPU Raman system was tested using anthracene, which has a relatively high Raman signal among the samples. The spectrum was obtained five times under the same condition of 400 Hz, duty ratio of 10%, and an integral time of 10,000 ms. After each measurement, the OPU Raman system was powered off, and Raman spectra were measured at intervals of 10 min. Figure 8a illustrates the spectra measured after each interval, whereas Fig. 8b provides a graph of the deviation (absolute value) of the position and intensity changes between spectra for the main peak, that is, 1,402 cm^−1^. The maximum deviation of the peak position is measured within ± 3 cm^−1^, whereas the peak intensity is measured within ± 6%. Considering the resolution of the commercial Raman spectrometer at 1 nm (10 cm^−1^), the OPU Raman system displays reasonable reproducibility.

The total cost used to manufacture the OPU Raman system is calculated as approximately 1,100 US$ or less when using a function generator as power source or an optical density (OD) 6 Raman edge filter. The cost was lower at approximately 930 US$ or less when a DC adapter is used to replace the power supply. If the OD 6 filter can be replaced with the OD 4 filter, then the costs are expected to reach approximately 730 US$. Based on the results, the OPU Raman system is expected to be widely built and utilized even in an educational research environment where budget is largely insufficient.

In summary, for the development of the Raman spectrometer under 1,000 US$, the study explored a Raman spectrometer consisting of the commercial OPU and a pulsed DC power. As a result of the experiment, Raman spectra with reasonable resolutions and indicated error rates of up to 11 cm^−1^ were obtained from the liquid and solid samples. In addition, the focal length of the OPU objective lens demonstrated controllability, and Raman signal intensity can be increased by controlling the voltage applied to the coil through a variable resistance adjustment. When the pulsed DC signal is used as power, the LD wavelength tended to move to a longer wavelength with the increase in the duty ratio. The higher the frequency at the same duty ratio, the weaker the intensity of the background signal compared with the Raman signal. The reproducibility of the OPU Raman spectrometer showed a maximum deviation in the peak position within ± 3 cm^−1^ with a peak intensity of ± 6%. This result showed excellent properties even when compared with a commercial Raman system using a 1 nm (10 cm^−1^) spectrometer. In terms of cost, the proposed system can be produced within a range of 730 US$ to 1,100 US$. Thus, the OPU Raman spectrometer is expected to be widely used in a low-budget education and research environment.

## Methods

Except for the frame and electromagnet coils of the OPU (KSS-213C), other parts, such as LD, circuit connections, covers, and a beam splitter, were removed. Subsequently, a 520 nm LD, collimator lens, Raman edge filter, and reflective mirror were bonded to the OPU frame. A 3D printer (CreatBot F160) was used to build the component holder or connections, an OPU Raman support, and sample holder (see CAD design in Supplementary Information). Finally, the power source for adjusting the focal length was connected, and the optical fiber was connected to a spectrometer to complete it. The components used for the fabrication of the OPU Raman were a 520-nm wavelength LD (PL 520, 50 mW, OSRAM), Raman edge filter with OD 6 (Ø12.5 mm, 537.3 nm, Raman edge filter, Edmund Optics), optical fiber (SMA-type, 200-µm core diameter), and Ag dot mirror with a diameter of 3 mm formed on a 0.1-mm-thick glass substrate using a silver mirror reaction. For solid samples, a sample container with a glass cover was prepared using a 3D printer and a piece of slide glass. For liquid samples, the sample was placed in a small vial with a diameter of 1.2 cm (to minimize air bubbles during sampling) and measured through the glass surface of the bottom of the vial. Benzene (Samchun, 99.5%) and acetone (Daejung, 99.8%) as liquid samples and naphthalene (Daejung, 97%) and anthracene (Daejung, 97%) as solid samples were measured. The Raman signal was measured by changing the duty ratio (10% to 80%) and frequency (50–500 Hz) using a function generator (Owon, AG051F) as the power source for the LD. The optical spectrometer (R spectrometer, Thunder Optics) was calibrated based on the positions of 992 and 3,062 cm^−1^, and the characteristic Raman peaks of benzene using least squares fitting were built into the software and provided with the optical spectrometer. Subsequently, the wavelength of the LD was measured according to the duty ratio and frequency characteristics using an ultraviolet–visible spectrometer (K-mac, 2100 V). The programs used to build and measure the OPU Raman spectrometers are all freeware: FreeCAD (ver. 0.17, Open Source), Ultimaker Cura for 3D printers (ver. 3.1.0, Ultimaker BV), and MagicPlot Student (ver. 2.7.2, MagicPlot Systems, LLC, for the baseline correction of Raman spectra).

## Supplementary information

Supplementary file1 (DOCX 16053 kb)

Supplementary file2 (FCSTD 29 kb)

Supplementary file3 (FCSTD 34 kb)

Supplementary file4 (FCSTD 27 kb)

Supplementary file5 (FCSTD 19 kb)

Supplementary file6 (FCSTD 23 kb)

Supplementary file7 (FCSTD 14 kb)

Supplementary file8 (FCSTD 14 kb)
