# Supplementary material for: Fabricating a Raman spectrometer using an optical pickup unit and pulsed power
Source: Sci Rep. 2020 Jul 16;10:11692. doi: 10.1038/s41598-020-68650-7 (PMC7366627; doi:10.1038/s41598-020-68650-7)
Supplement: Supplementary file 1 — Supplementary file1 (DOCX 16053 kb) [file 41598_2020_68650_MOESM1_ESM.docx]

**Supplementary Information**

**Fabricating a Raman Spectrometer Using an Optical Pickup Unit and Pulsed Power**

Young Chai Cho, Sung Il Ahn^*^

Department of Chemistry Education, Graduate Department of Chemical Materials, Institute for Plastic Information and Energy Materials, Pusan National University, Busandaehakro 63-2, Busan 46241, Republic of Korea

**1. Manufacturing process of the OPU Raman system**

**1-1. Disassembly of OPU (KSS-213C)**

**1-2. Beam splitter replacement**

**1-3. Fabrications of interconnectors using a 3D printer**

**1-4. Connecting the external power of the lens coil**

**2. Baseline correction method of the Raman spectra**

**1. Manufacturing process of the OPU Raman system**

The CAD files designed in this study have been uploaded as a supplementary file in the home page of the journal.

**1-1. Disassembly of OPU (KSS-213C)**

**Figure S1**. (a) Image of KSS-213C OPU. (b) Images of disassembled OPU (front and back).

**1-2. Beam splitter replacement**

**Figure S2**. Replacement of the OPU beam splitter with Ag dot mirror

In this process, setting the position of the coated mirror dot to match the laser beam path is important. The dot mirror surface must be secured to exactly touch the laser beam path. In this study, the laser power was adjusted to be as low as possible, and the position was adjusted by viewing the light path and gradually adjusting the position.

**1-3. Fabrications of interconnectors using a 3D printer**

**a. LD and its holder**

**Figure S3**. LD and its holder attachment. (a) Adhesion position of LD. (b) LD image used in the study. (c) Combined LD and its holder.

Due to the nature of the 3D printer, a slight difference in size may be noted between the design and finished product due to the operating environment or material. Therefore, size correction may be necessary depending on the production environment. The design of this study is basically based on the specifications of KSS-213C. However, a fine correction is noted in the actual design size.

**b. Raman Edge filter and its holder**

**Figure S4**. Attached Raman edge filter combined with a holder. (a) Printed Raman edge filter holder. (b) Raman edge filter and its holder attached to the disassembled OPU.

Attaching this part on the optical path that can receive as much scattered light as possible with the filter is extremely important. If the optical path alignment is not properly performed, then a problem could be that only simple reflected light is measured instead of the desired Raman scattering spectrum. In this study, the adjustment was repeated and aligned, whereas the position was gradually changed using a low-power laser.

**c. Fiber optic adapter combined with collimator lens**

**Figure S5.** Attachment of the fiber-optic adapter combined with a collimator lens. (a) Fiber-optic adapter combined with collimator lens. (b) Image of connecting the adapter with a fiber optic. (c) Image of the attached adapter to the OPU in Figure S4b.

After measuring the focal length of the lens, the adapter was designed, such that the distance between the optical fiber and lens remains proportional to the focal length of the lens, which was attached to the adapter using an adhesive or double-sided tape.

**d. OPU Raman fixed frame and sample fixed frame**

**Figure S6**. Images of the OPU Raman and sample fixed frames. (a) Attached image of the OPU Raman fixed frame to the OPU in Figure S5c. (b) Attached image of the sample fixed frame to the OPU in (a).

**e. Light-block box and the sample bottle for solid**,

**Figure S7.** Images of the light-block box and sample bottle for the solid. (a) OPU Raman covered with a light-block box. (b) Sample bottle containing the solid sample.

**1-4. Connecting the external power of the lens coil**

**Figure S8.** Lens coil connected by wires

**2. Baseline correction method of OPU Raman spectrum**

Raman spectra were acquired after baseline fitting using MagicPlot Student (ver. 2.7.2, MagicPlot Systems, LLC). Figure S9 describes the correction process. First, the Gaussian and parabola fitting curves were created in the program, and the baseline was obtained, as shown in Figure S9. Then, the original graph of sample A is modified using the baseline. Adding more fitting curves to the Gaussian or parabola curve can increase baseline precision.

**Figure S9.** Baseline correction process of the sample. (a) Baseline created using the Gaussian and parabola curves of the curve fitting program. (b) Baseline corrected spectrum after subtracting the “fit–sum” baseline in (a).
